# Development of a novel defined minimal medium for *Gluconobacter oxydans* 621H by systematic investigation of metabolic demands

**DOI:** 10.1186/s13036-022-00310-y

**Published:** 2022-11-21

**Authors:** Svenja Battling, Johannes Pastoors, Alexander Deitert, Tobias Götzen, Lukas Hartmann, Eliot Schröder, Stanislav Yordanov, Jochen Büchs

**Affiliations:** AVT – Biochemical Engineering, RWTH Aachen University, Forckenbeckstraße 51, 52074 Aachen, Germany

**Keywords:** *Gluconobacter oxydans*, Minimal medium, Synthetic media, 5-ketofructose, μRAMOS, Media development, Auxotrophy

## Abstract

**Background:**

Historically, complex media are used for the cultivation of *Gluconobacter oxydans* in industry and research. Using complex media has different drawbacks like higher costs for downstream processing and significant variations in fermentation performances. Synthetic media can overcome those drawbacks, lead to reproducible fermentation performances. However, the development of a synthetic medium is time and labour consuming. Detailed knowledge about auxotrophies and metabolic requirements of *G. oxydans* is necessary. In this work, we use a systematic approach applying the in-house developed μRAMOS technology to identify auxotrophies and develop a defined minimal medium for cultivation of *G. oxydans fdh*, improving the production process of the natural sweetener 5-ketofructose.

**Results:**

A rich, defined synthetic medium, consisting of 48 components, including vitamins, amino acids and trace elements, was used as a basis for medium development. In a comprehensive series of experiments, component groups and single media components were individually omitted from or supplemented to the medium and analysed regarding their performance. Main components like salts and trace elements were necessary for the growth of *G. oxydans fdh*, whereas nucleotides were shown to be non-essential. Moreover, results indicated that the amino acids isoleucine, glutamate and glycine and the vitamins nicotinic acid, pantothenic acid and p-aminobenzoic acid are necessary for the growth of *G. oxydans fdh*. The glutamate concentration was increased three-fold, functioning as a precursor for amino acid synthesis. Finally, a defined minimal medium called ‘*Gluconobacter* minimal medium’ was developed. The performance of this medium was tested in comparison with commonly used media for *Gluconobacter.* Similar/competitive results regarding cultivation time, yield and productivity were obtained. Moreover, the application of the medium in a fed-batch fermentation process was successfully demonstrated.

**Conclusion:**

The systematic investigation of a wide range of media components allowed the successful development of the *Gluconobacter* minimal medium. This chemically defined medium contains only 14 ingredients, customised for the cultivation of *G. oxydans fdh* and 5-ketofructose production. This enables a more straightforward process development regarding upstream and downstream processing. Moreover, metabolic demands of *G. oxydans* were identified, which further can be used in media or strain development for different processes.

**Supplementary Information:**

The online version contains supplementary material available at 10.1186/s13036-022-00310-y.

## Background

In the transition to a sustainable bioeconomy, reducing the costs of biosynthesis and subsequent product recovery is one key challenge in order to become and remain competitive with conventional petrochemical production processes. One promising approach for cost reduction is the use of synthetic media instead of conventional complex media containing e.g. yeast extract or peptone. Although the cost of synthetic media components is not necessarily smaller than the cost of complex components [1], the application of synthetic media can significantly reduce the cost of the bioprocess indirectly. Complex components often entail lot-to-lot variations that can strongly influence fermentation performance [1–3]. This can directly affect product quality and, therefore, reduce product yields. In addition, understanding and mass balancing of the microbial process are easier in a chemically defined medium than in a scarcely defined complex medium. Moreover, complex media components in fermentations can complicate the subsequent downstream processing, often demanding additional purification steps, in comparison to synthetic fermentation processes [1, 4–6]. However, the application of synthetic media requires detailed knowledge about the auxotrophies and metabolic requirements of the cultivated microorganism.

*Gluconobacter oxydans* belongs to the acetic acid family and is a strictly aerobic microorganism. Its metabolism is of particular interest, as glycolysis and tricarboxylic acid cycle are incomplete, missing the enzymes phosphofructokinase and succinate dehydrogenase, respectively [7, 8]. Therefore, the pentose phosphate pathway becomes the most important catabolic route in *G. oxydans*. Despite the unusual metabolism, *G. oxydans* is well known for its ability to incompletely oxidise various alcohols and carbohydrates [9, 10]. The most prominent industrial application of *G. oxydans* is vitamin C production. *G. oxydans* is used for the regioselective oxidisation of D-sorbitol to L-sorbose in the conventional Reichstein process [8, 11]. Current research focuses on replacing chemical synthesis steps in this process with biotechnological alternatives, for example using genetically modified *G. oxydans* strains [12, 13].

In 2018, a production process of the potential sweetener 5-keto-d-fructose (5KF) was reported by Herweg et al. [14]. Product titres of up to 489 g_5KF_/L and a product yield of up to 0.98 g_5KF_/g_fructose_ were reached [14, 15]. 5KF shows a similar sweet taste quality as fructose and can be found in musts and wine [16], thus, being a new candidate for natural sweeteners. The production process is based on a complex medium containing yeast extract and fructose as substrate. Membrane-bound dehydrogenases have been shown to be responsible for the oxidation reactions leading to very high product yields [8, 17]. In contrast, only low amounts of biomass are produced. Genomic sequencing showed that *G. oxydans* contains all metabolic pathways for the de novo synthesis of nucleotides, amino acids and most vitamins [7].

Although its genome has been completely sequenced [18], complex media are still commonly used in processes with *G. oxydans* [8, 14, 15, 19, 20]. Various research efforts have been made to determine the nutritional requirements of *Gluconobacter* strains using synthetic media and semi-synthetic media [21–25]. The influence of various vitamins and amino acids on the growth of *G. oxydans* has been investigated [21, 23, 26]. While growth and product formation were generally possible in synthetic media [27, 28], complex media have not yet been substituted in production processes [14, 15].

The development of a minimal medium and the investigation of growth demands is labour and time consuming, as previously mentioned publications have shown. Usually, for each ingredient, growth curves were determined by manually taking samples over time and measuring the optical density [23, 25, 26]. As an alternative, Müller et al. [29] developed an efficient and rapid method for the identification of ingredients necessary for growth, using a respiration activity monitoring system (RAMOS) for microtitre plates (μRAMOS).

In this work, we transferred the method of Müller et al. [29] on the cultivation of *G. oxydans* 621H Δ*hsdR* pBBR1-p264-FDH-ST (*G. oxydans fdh)*, to develop a minimal medium for 5KF production. The aim was to establish an efficient 5KF production process based on a chemically defined medium with a limited number of components. Meanwhile, cultivation times, biomass formation and product yields should be comparable to the process in the established complex medium. For this purpose, a rich, defined, synthetic medium by Poolman et al. [30] was modified, and its components were systematically investigated regarding their influence on the cultivation performance of *G. oxydans fdh* [29]. In a comprehensive series of experiments, component groups and single media components were individually omitted from or supplemented to the medium and respiration of the cultivations was monitored using the in-house developed μRAMOS-technology. The gained information was then used to identify and select important media components for a reduced minimal medium. As a result, costs can be reduced, in comparison to the rich complete Poolman medium.

## Materials and methods

The organism used in this work was the strain *G. oxydans* 621H Δ*hsdR*, which contains the plasmid pBBR1-p264-FDH-ST (*G. oxydans fdh)*. The expression vector was developed by Siemen et al. [15] at the Institute of Microbiology and Biotechnology at the University of Bonn for the heterologous overproduction of the membrane-bound enzyme fructose dehydrogenase (FDH) [27, 31]. The organism has a natural resistance to cefoxitin, and the plasmid contains a gene for kanamycin resistance. For strain maintenance, stocks containing 200 g/L glycerol were used and stored at − 80 °C.

### Media composition

The *Gluconobacter* complex medium for pre- and main cultivations contained 5 g/L yeast extract (Karl Roth GmbH, Karlsruhe, Germany or BD Biosciences, Heidelberg, Germany), 2.5 g/L MgSO_4_ ∙ 7H_2_O, 1 g/L (NH_4_)_2_SO_4_, 1 g/L KH_2_PO_4_ (Table 1). The initial pH was adjusted to 6 with KOH, and the medium was sterile filtered [33].

**Table 1:** Composition of *Gluconobacter* complex medium [33], complete Poolman medium modified by Müller et al. [29, 30, 32], *Gluconobacter* minimal medium (GMM) and modified Ameyama medium [27]

Ingredients	***Gluconobacter*** complex medium [g/L]	Complete Poolman [g/L]	GMM [g/L]	Ameyama [g/L]
**Main ingredients**				
Yeast extract	5			
K_2_HPO_4_				0.5
KH_2_PO_4_	1	1	1	0.5
NaCl				0.01
(NH_4_)_2_SO_4_	1	1	1	
MgSO_4_ ∙ 7H_2_O	2.5	2.5	2.5	0.2
**Amino acids**				
Alanine		0.24		
Arginine		0.125		
Aspartate		0.42		
Cysteine		0.13		
Glutamate		0.5	1.5	6
Glycine		0.175	0.175	
Histidine		0.15		
Isoleucine		0.21	0.21	
Leucine		0.475		
Lysine ∙ H_2_O		0.495		
Methionine		0.125		
Phenylalanine		0.275		
Proline		0.675		
Serine		0.34		
Threonine		0.225		
Tryptophan		0.05		
Tyrosine		0.25		
Valine		0.325		
**Vitamins**				
Ascorbic acid		0.5		
Biotin		0.003		
Folic acid		0.001		
Nicotinic acid		0.001	0.001	0.0004
Ortoric acid		0.005		
P-aminobenzoic acid		0.01	0.01	0.0001
Pantothenic acid		0.001	0.001	0.0004
Pyridoxamine ∙ 2HCl		0.005		
Pyridoxine ∙ HCl		0.002		
Riboflavin		0.001		
Thiamine ∙ HCl		0.001		0.0004
Vitamin B12		0.001		
**Nucleobases/nucleosides**				
Adenine		0.01		
Cytosine		0.073		
Guanine		0.01		
Inosine		0.005		
Thymidine		0.005		
Uracil		0.01		
Xanthine		0.01		
**Trace elements**				
CaCl_2_ ∙ 2H_2_O		0.007	0.007	
CoSO_4_ ∙ 7H_2_O		0.005	0.005	
CuSO_4_ ∙ 5H_2_O		0.004	0.004	
FeCl_2_		0.005	0.005	
FeCl_3_ ∙ 6H_2_O		0.005	0.005	
FeSO_4_ ∙ 7H_2_O				0.001
MnCl_2_		0.016	0.016	
MnSO_4_ ∙ 7H_2_O				0.001
(NH_4_)_6_Mo_7_O_24_ ∙ 4H_2_O		0.003	0.003	
ZnSO_4_ ∙ 7H_2_O		0.009	0.009	

The chemically defined Poolman minimal medium was modified by Müller et al. [29, 30, 32] and complemented with 2.5 g/L MgSO_4_ ∙ 7H_2_O, 1 g/L (NH_4_)_2_SO_4_ and 1 g/L KH_2_PO_4_. This medium will be referred to as complete Poolman medium in the following. The composition can be found in Table 1. MgSO_4_ ∙ 7H_2_O, (NH_4_)_2_SO_4_ and KH_2_PO_4_ were prepared separately, autoclaved and stored at room temperature. Amino acid, vitamin and nucleotide stock solutions were prepared separately and stored at 4 °C. If necessary, HCl was added to dissolve the components. The trace elements stock solution was stored at 4 °C containing all trace elements except FeCl_2_ and FeCl_3_ ∙ 6H_2_O. The iron stock solution was stored at − 20 °C. For cultivations, all components were mixed. The initial pH was adjusted to 6 with KOH and HCl. MgSO_4_ ∙ 7H_2_O, (NH_4_)_2_SO_4_ and KH_2_PO_4_ were added after adjusting the pH. Unless otherwise stated, all media components were sterile filtered using a 0.2 μm cut-off filter (VWR International GmbH, Darmstadt, Germany) and diluted in demineralised water.

The composition of the chemically defined Ameyama medium can be found in Table 1 [27]. The components were prepared as described for the Poolman medium.

Unless otherwise stated, all main cultivation media were supplemented with 50 μg/mL kanamycin and 60 g/L fructose. The pre-cultivation medium contained *Gluconobacter* complex medium (Table 1), supplemented with 80 g/L mannitol, 50 μg/mL kanamycin and 50 μg/mL cefoxitin.

### Cultivation conditions

Pre-culture cultivations were performed in unbaffled shake flasks using the Respiration Activity Monitoring System (RAMOS), developed at our chair [34, 35]. Commercial versions of the RAMOS device can be acquired from Kühner AG (Birsfeld, Switzerland) or HiTec Zang GmbH (Herzogenrath, Germany). Eight 250 mL flasks were equipped with an oxygen partial pressure sensor and differential pressure sensors, to determine the oxygen transfer rate (OTR), the carbon dioxide rate (CTR) and the respiratory quotient (RQ). The cultivations were performed with an initial filling volume of 10 mL, 350 rpm shaking frequency and 50 mm shaking diameter (Climo-Shaker ISF1-X, Kuhner, Birsfelden, Switzerland). Pre-cultures were inoculated with 100 μL glycerol stock cell suspension and cultivated at 30 °C for 11 h to 19 h. The main culture was inoculated with an optical density measured at 600 nm (OD_600_) of 0.1 from the pre-culture. Pre-culture cells were centrifuged for 3 minutes at 16,214 *g* and room temperature and resuspended in main culture medium.

Main cultivations were also performed in 48-well round well microtitre plates (MTP, m2p-labs, Baesweiler, Germany) using a μRAMOS device developed at our chair [36]. The μRAMOS enables measurement of the oxygen partial pressure in every individual well of an MTP. The measuring principle is based on the oxygen-dependent emission of fluorescence sensors. MTP cultivations were performed with an initial filling volume of 500 μL, 1000 rpm shaking frequency and 3 mm shaking diameter (Climo-Shaker ISF1-X, Kuhner, Birsfelden, Switzerland). The MTPs were covered with a gas-permeable Polyolefin sealing foil (HJ-Bioanalytik GmbH, Erkelenz, Germany), to reduce evaporation and prevent contaminations.

Fermentation experiments were performed in two different fermenters: Fermenter A: 2 L Sartorius BIOSTAT® Bplus stirred tank reactor (Sartorius, Goettingen, Germany). The dissolved oxygen tension (DOT) was measured using a VisiFerm™ DO 225 pO_2_ sensor (Hamilton, Hoechst, Germany) and maintained at 30% by variation of the agitation speed (500 rpm – 1500 rpm). A DASGIP G4 exhaust gas analyser (DASGIP, Eppendorf, Jülich, Germany) was used to determine the oxygen and carbon dioxide concentrations used for OTR, CTR and RQ calculations. Fermenter B: 2 L New Brunswick™ BioFlo®/CelliGen® benchtop bioreactor (Eppendorf, Germany). The DOT was measured using a Clark electrode (Mettler Toledo, Gießen, Germany) and maintained at 30% by variation of the agitation speed (500 rpm – 1350 rpm). A Rosemount X-Stream NGA 2000 exhaust gas analyser (Emerson, St. Louis, Missouri, USA) was used to determine the oxygen and carbon dioxide concentrations used for OTR, CTR and RQ calculations. Fermenters A and B: Both fermenters were equipped with one six-blade Rushton turbine for batch and two six-bladed Rushton turbines for extended-batch cultivations and four baffles. The pH value was measured using a pH sensor (EasyFerm Plus K8 200, Hamilton, Hoechst, Germany). 0.5 mL antifoam agent Plurafac LF 1300 (BASF, Ludwigshafen, Germany) was added at the beginning of each experiment and when needed to prevent foaming. Fermentations were started with an initial filling volume of 1 L and an aeration rate of 1 L/min. The feed solution consisted of 760 g/L or 825 g/L fructose and was sterile filtered. A peristaltic pump (Reglo analog ISM830, ISMATEC, Wertheim, Germany) was used for feeding. During fermentations, the weight of the fructose feed reservoir was recorded and used to determine the applied fructose feeding rates. pH was controlled at 5 using 10 M KOH after the initial batch phase. Volume change by KOH titration, sampling and fructose feeding was considered for mass balancing and all calculations.

### Offline analyses

The OD_600_ was measured photometrically in disposable cuvettes (UV cuvettes, semi-micro, Brand, Wertheim, Germany) using a spectrophotometer (Genesys 20, Thermo Scientific, Darmstadt, Germany). Since a linear correlation for OD_600_ and cell mass according to the Lambert-Beer law is only viable for an OD_600_ between 0.1 and 0.3, samples were diluted using 0.9% (w/v) NaCl, if necessary. The pH was measured using a HI221 Basic pH-meter (Hanna Instruments Deutschland GmbH, Vöhringen, Germany), calibrated daily with two standard buffer solutions at pH 4 and 7. The determination of fructose and 5KF concentrations was carried out via high-performance liquid chromatography (HPLC). A HPLC system Shimadzu Prominence LC-20 (Duisburg, Germany) was used. The HPLC was equipped with a precolumn Organic Acid Resin (40 × 8 mm, CS-Chromatographie Service, Langerwehe, Germany), the separating column Organic Acid Resin (250 × 8 mm, CS-Chromatographie Service, Langerwehe, Germany), and a refraction index detector RID-20A (Shimadzu, Duisburg, Germany). The flow rate of the mobile phase (5 mM H_2_SO_4_) was set to 0.8 mL/min with a column temperature of 30 °C. Fructose and 5KF standards in concentrations between 0.064 g/L and 10 g/L were used to prepare the standard curves. For HPLC measurement, fermentation samples were centrifuged for 3 minutes at 16,214 *g* and room temperature. The supernatant was diluted with deionised water, if necessary, sterile filtered (0.2 μm syringe filter, Whatman™, GE Healthcare, Freiburg, Germany) and heated to 60 °C for 60 min (avoiding double peaks, probably caused by the existence of 5KF in an equilibrium of the keto and the germinal diol form [14]). Yields were calculated by dividing the produced 5KF by the total fructose concentration and are indicated in g/g.

## Results and discussion

### Cultivation of *G. oxydansfdh* in defined minimal medium and influence of different component groups

For the first experiment, the complete Poolman medium was divided into five component groups: nucleotides, trace elements, salts, amino acids and vitamins (Table 1). While the other component groups originated from the medium published by Poolman et al. [30], the component group of salts consist of (NH_4_)_2_SO_4_, KH_2_PO_4_ and MgSO_4_∙7H_2_O. These components are also included in the *Gluconobacter* complex medium and supply the organism with phosphate, nitrogen and magnesium. Therefore, they are expected to be essential for the media investigated in this work.

**Fig. 1:**
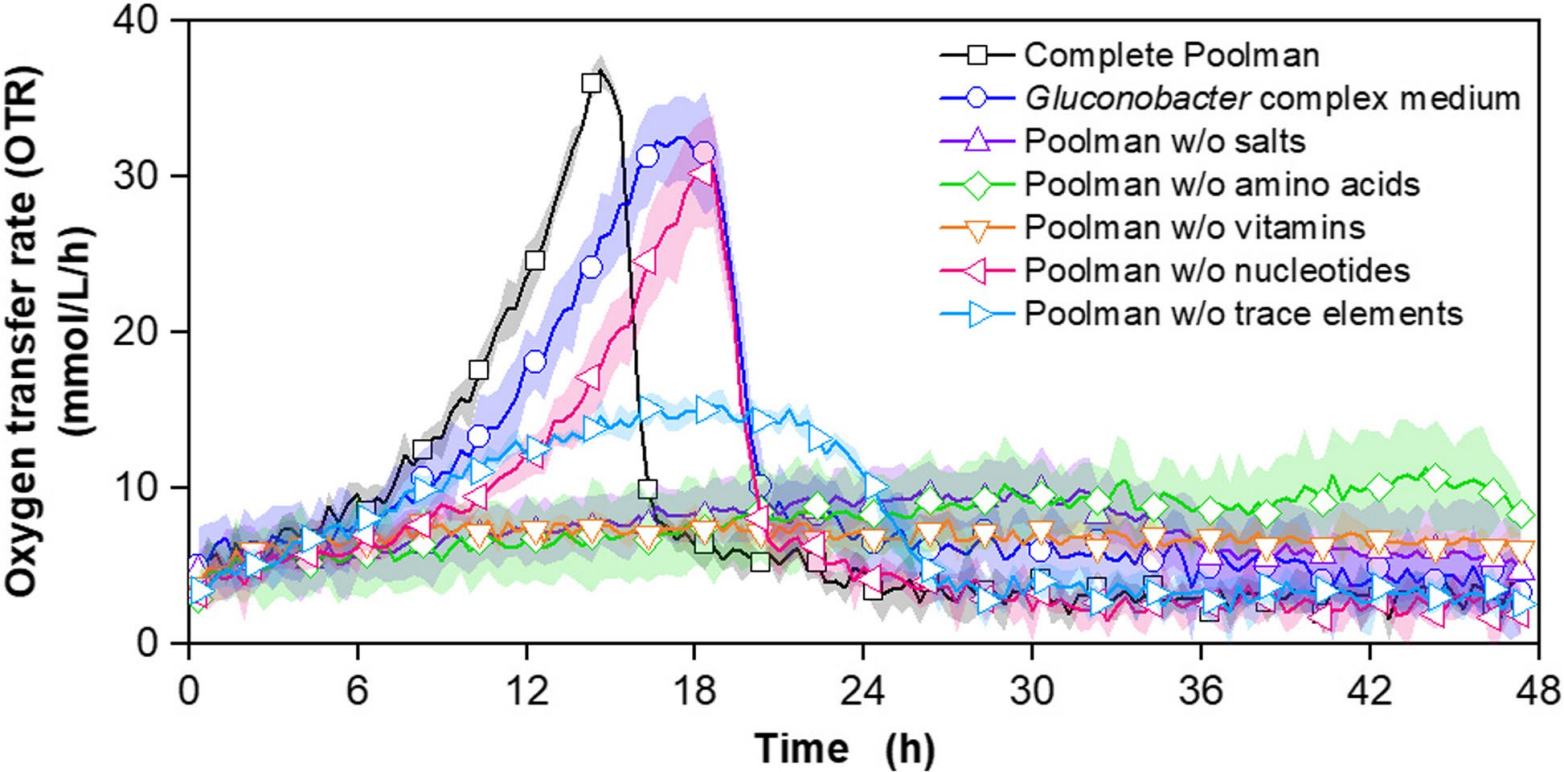
Cultivation of *G. oxydans* 621H Δ*hsdR* pBBR1p264-FDH-ST in a μRAMOS device with 60 g/L fructose and different media. Depicted is the oxygen transfer rate (OTR). Cultivations were performed at 30 °C, 1000 rpm, V_L_ = 500 μL in a 48-well round well microtitre plate at a shaking diameter of 3 mm, initial pH value: 6. Media: Complete Poolman medium as reference (black), *Gluconobacter* complex medium containing 5 g/L yeast extract, 2.5 g/L MgSO_4_ ∙ 7H_2_O, 1 g/L (NH_4_)_2_SO_4_ and 1 g/L KH_2_PO_4_ (dark blue), Poolman without salts (light blue), without amino acids (green), without vitamins (orange), without nucleotides (pink), without trace elements (purple). Mean values of at least 4 replicates are shown. The shadows around the curves indicate the standard deviation. For clarity, only every fifth measuring point is marked as a symbol

The growth of *G. oxydans fdh* in the complete Poolman medium was compared to growth in the established *Gluconobacter* complex medium with 60 g/L fructose. To gain a first impression of which component groups are essential for the growth of *G. oxydans fdh*, five additional cultivations in the complete Poolman medium, each lacking one of the five component groups, were conducted in parallel (Fig. 1).

*G. oxydans fdh* showed the typical OTR course in the *Gluconobacter* complex medium [14, 37]. The OTR increased exponentially up to a peak at approx. 32 mmol/L/h after 16 hours, followed by a sharp decline to 5 mmol/L/h, before the OTR decreased slowly over the following hours. The cultivation in complete Poolman medium showed a comparable OTR course, with a shifted OTR maximum of 36 mmol/L/h after 14 hours. Consequently, the growth of *G. oxydans fdh* is not limited, when cultivated in the rich, synthetic medium, but slightly improved, compared to the cultivation in *Gluconobacter* complex medium. Cultivations in Poolman medium without salts, amino acids, vitamins and trace elements showed significantly reduced OTRs. In contrast, the cultivation in the Poolman medium without nucleotides showed a similar OTR course as observed for *Gluconobacter* complex medium. In conclusion, supplementation of nucleotides improves growth in *G. oxydans fdh*, yet it is not essential. Since pathways for the synthesis of all nucleotides are included in the genome of *G. oxydans* [18] and nucleotides are not commonly supplemented in synthetic media, the entire component group will not be included in the final minimal medium.

An increase in OTR up to 15 mmol/L/h after 18 hours can be observed during the cultivation without trace elements. Trace elements are essential for microbial growth and should, therefore, be supplemented in synthetic media [38]. They are commonly included in the form of trace element solutions, without detailed consideration of the single components [39–41]. The conversion of fructose to 5KF is stoichiometrically linked to oxygen consumption and, thus, visible in the OTR [14]. In the experiment shown in Fig. 1, respirational activity can still be observed, which is due to the transfer of small amounts of trace elements from the pre-culture and the highly active FDH [14, 15]. However, the reduced concentration of trace elements still impaired growth. Consequently, the trace element solutions of the complete Poolman medium remained unchanged and were used for further experiments.

The cultivations lacking salts, amino acids or vitamins showed only slight increases in OTR and significantly longer cultivation times. Hence, all component groups except nucleotides include at least one component crucial for *G. oxydans fdh.*

Nitrogen, phosphate and magnesium have important functions in an organism’s metabolism and are included in most synthetic media [39, 40]. Nitrogen is the fourth most common element in the biomass of *G. oxydans* [42] and is essential for its optimal growth [43, 44]. Phosphate plays a crucial role in energy metabolism [45] and the pentose phosphate pathway [46], which is of particular importance for oxidising sugars in *Gluconobacter* strains [22]. Magnesium is important for cell proliferation in most organisms by stimulating DNA and protein synthesis [47, 48]. The influence of different nitrogen sources will be further discussed later in this work. KH_2_PO_4_ and MgSO_4_∙7H_2_O will be used in unchanged concentration in the complete Poolman medium.

The complete Poolman medium contains 18 different amino acids as well as 12 vitamins. Since the cultivations using the Poolman medium without amino acids or vitamins showed very limited growth, at least one amino acid and vitamin are essential for the growth of *G. oxydans fdh*. As a result, five amino acids groups and four vitamin groups (Table 2) were established, according to the classification by Müller et al. [29]. It is based on metabolic pathways described by Akashi and Gojobori [29, 42]. Single amino acids and vitamins were further investigated in the following.

**Table 2:** Group of amino acids and vitamins

**Group #**	**Amino acids**				
1	Aspartate	Isoleucine	Methionine	Threonine	
**2**	Arginine	Glutamate	Histidine	Proline	
**3**	Cysteine	Glycine	Serine		
**4**	Phenylalanine	Tryptophan	Tyrosine		
**5**	Alanine	Leucine	Lysine	Valine	
**Group #**	**Vitamins**				
**6**	Nicotinic acid	Pantothenic acid	P-Aminobenzoic acid		
**7**	Pyrodoxamine	Pyridoxine	Folic acid	Ortoric acid	Riboflavin
**8**	Ascorbic acid	Biotin			
**9**	Thiamine	Vitamin B12			

### Investigation of the influence of amino acids on the growth of *G. oxydansfdh*

The influence of amino acids on the growth and metabolism of *Gluconobacter* species has been intensively studied for decades. Since 1953, supplementation of amino acids has been reported to support growth in various *Gluconobacter* species [23, 25, 43]. Sainz et al. [28] showed that the optimal composition and concentration of amino acids deviate between different *Gluconobacter* species. Gosselé et al. [24] stated that no single amino acid is essential for the growth of most *Gluconobacter* strains, as growth was not decreased by the omission of single amino acids in their investigations. Consequently, a detailed study of the influence of amino acids on the growth of *G. oxydans fdh* in the Poolman medium was carried out in this work. In a series of experiments, the five amino acid sub-groups were individually omitted from the complete Poolman medium. When an influence of the omission on growth was observed, the single amino acids of the investigated group were added again. The resulting cultivations were compared to the cultivation without the concerning sub-group, to evaluate the influence of the single components. The composition of the amino acid sub-groups can be found in Table 2.

**Fig. 2:**
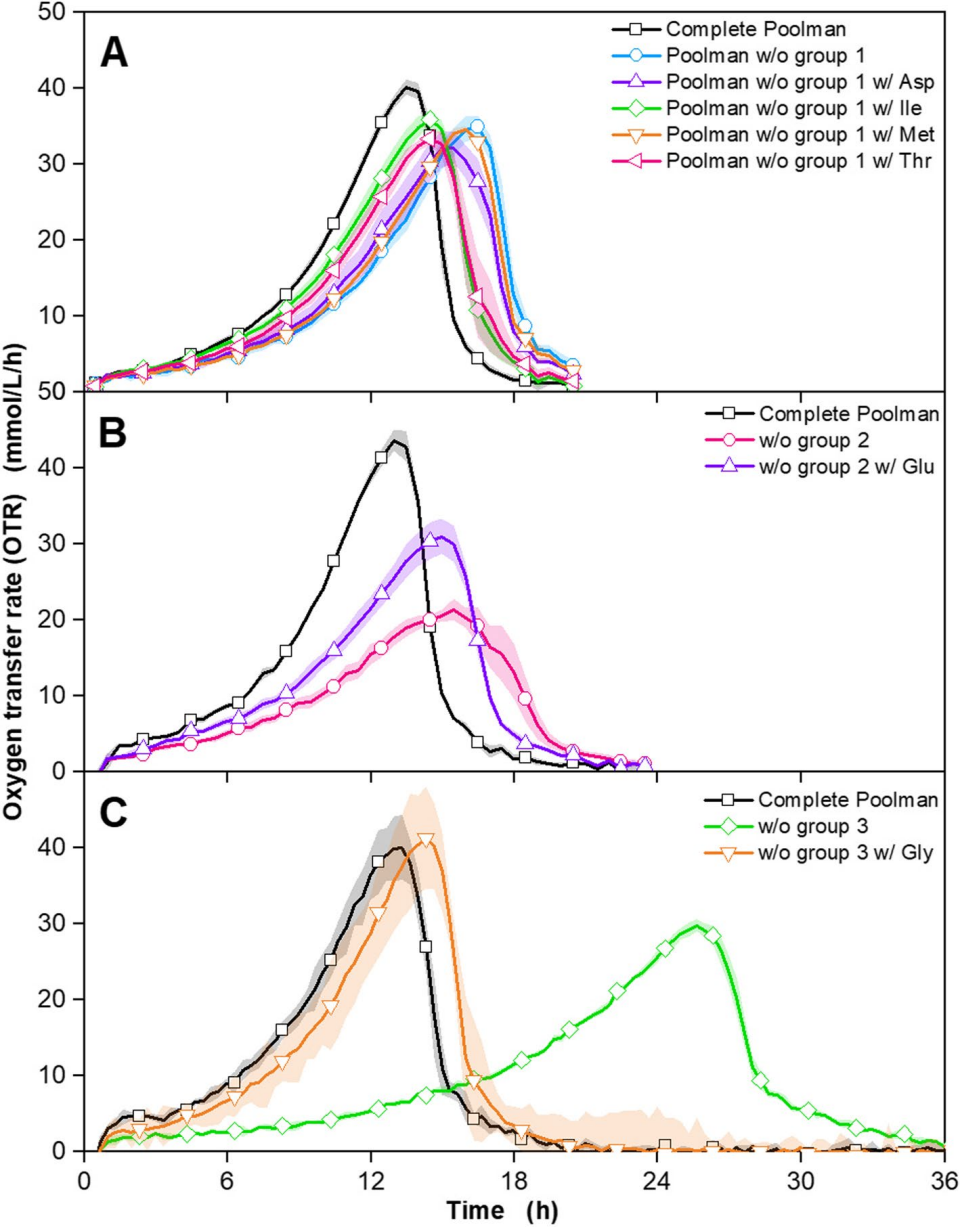
Investigation of auxotrophic deficiencies of *G. oxydans* 621H Δ*hsdR* pBBR1p264-FDH-ST in Poolman medium regarding the amino acids of group 1 (Asp, Ile, Met, Thr), group 2 (Arg, Pro, His, Glu) and group 3 (Cys, Gly, Ser). Depicted is the oxygen transfer rate (OTR). Cultivation of *G. oxydans* 621H Δ*hsdR* pBBR1p264-FDH-ST in a μRAMOS device with 60 g/L fructose, lacking amino acids from group 1 (aspartate, isoleucine, methionine, threonine), group 2 (arginine, glutamine, histidine, proline) and group 3 (cysteine, serine, glycine). Three independent cultivations (**A, B** and **C**) were performed at 30 °C, 1000 rpm, V_L_ = 500 μL in a 48-well round well microtitre plate at a shaking diameter of 3 mm, initial pH value: 6. Media: Complete Poolman medium as reference (black) and **A** without group 1 (light blue), without group 1 with aspartate (purple), without group 1 with isoleucine (green), without group 1 with methionine (orange), without group 1 with threonine (pink), **B** without group 2 (pink) and without group 2 with glutamate (purple), **C** without group 3 (green) and without group 3 with glycine (orange). Mean values of at least 6 replicates are shown. The shadows around the curves indicate the standard deviation. For clarity, only every fifth measuring point is marked as a symbol

The results for groups 1–3 are displayed in Fig. 2, as omitting groups 4 and 5 did not negatively influence the growth of *G. oxydans fdh* (Fig. S1). Omitting group 1 from the complete Poolman medium led to a slightly delayed increase in OTR and a decreased peak height, compared to the complete Poolman medium.

Figure 2 A). The addition of aspartate or methionine showed no positive influence on the respiration of *G. oxydans fdh*. Adding isoleucine or threonine, however, reduced the time until the OTR peak is reached by 2 hours. This effect was slightly stronger for the addition of isoleucine. Since respiration remains similar to the complete Poolman medium, it can be concluded that no amino acid in group 1 is absolutely essential for the growth of *G. oxydans fdh*. This is not surprising, as the components of group 1 did not receive much attention in research on *Gluconobacter* strains so far [21, 22]. Aspartate has been reported to be an important nutrient for acetic acid bacteria [44], while some *Gluconobacter* strains are able to deaminate threonine [24]. However, since isoleucine supplementation led to accelerated growth, isoleucine is included in the medium for further experiments.

Leaving out group 2 from the Complete Poolman medium reduced the respirational activity significantly (Fig. 2 B). The maximal OTR of 20 mmol/L/h was reached after 17 hours, compared to the OTR of the complete Poolman medium, which increased up to 45 mmol/L/h after 13 hours. The addition of glutamate increased the OTR peak height and reduced the time of the OTR peak to 32 mmol/L/h after 16 hours. Especially glutamate has been found to improve the growth of different acetic acid bacteria [22, 45], while Sainz et al. [28] described histidine as a sufficient sole nitrogen source for *G. oxydans* 621H. Like all amino acids, glutamate can be synthesised de novo by *G. oxydans* [18]. In the biosynthesis of other amino acids, it is an important donor of amino groups as well as a precursor. Moreover, it acts as an acceptor molecule for inorganic nitrogen in the ammonium assimilation of the cell [46]. In conclusion, glutamate is crucial for efficient growth and is, thus, included in different media for the cultivation of *G. oxydans* [22, 23, 47]. It is not surprising that supplementation of glutamate improves the cultivation of *G. oxydans fdh,* as evidenced by the observed increase in respirational activity (Fig. 2 B). Adding the other single amino acids of group 2 led to a similar effect (Fig. S1A). Glutamate, however, improved the cultivation the most and is, therefore, chosen to remain in the medium, to reach growth similar to the *Gluconobacter* complex medium with as few components as possible.

The cultivation without the addition of group 3 showed a significantly delayed increase in OTR, compared to the complete Poolman medium, reaching 30 mmol/L/h after 26 hours (Fig. 2 C). When glycine was added, the OTR progressed nearly identical to the complete medium with only a slight delay in reaching the maximum OTR. An almost identical observation was made for serine supplementation (Fig. S1B). Wethmar et al. [21] reported that serine was one of two amino acids required for the growth of *G. oxydans* in their work. In contrast, Tachiki et al. [48] described that glycine inhibited the glutamate-glutamine metabolism in *G. suboxydans*. According to the genome sequence of *G. oxydans* by Prust et al. [18], serine acts as a precursor for glycine production. Yoshitake et al. [49] described the microbial conversion of glycine to serine. In this context, the nearly identical results of serine and glycine addition to the medium are very plausible, and one of both components should remain in the medium. Since the cultivation with glycine showed a slightly shorter cultivation time (Fig. S1B), glycine was chosen as the remaining media component of group 3 for further experiments.

The amino acids in group 4 have been reported to play a role in the activity of single enzymes of *G. oxydans* [50, 51], while valine from group 5 has been reported to have an inhibitory effect on the growth of *Acetobacter suboxydans* [52]. Because of their limited influence on the growth of *G. oxydans fdh*, groups 4 and 5 were decided to be discarded from the medium (Fig. S1C). After investigation of all amino acids, only isoleucine, glutamate and glycine remain in the medium for further experiments. Since the nitrogen content of the media is strongly affected by the omission of the majority of amino acids, the influence of different nitrogen sources is investigated in the next step.

### Investigation of nitrogen supply for the cultivation of *G. oxydansfdh*

**Fig. 3:**
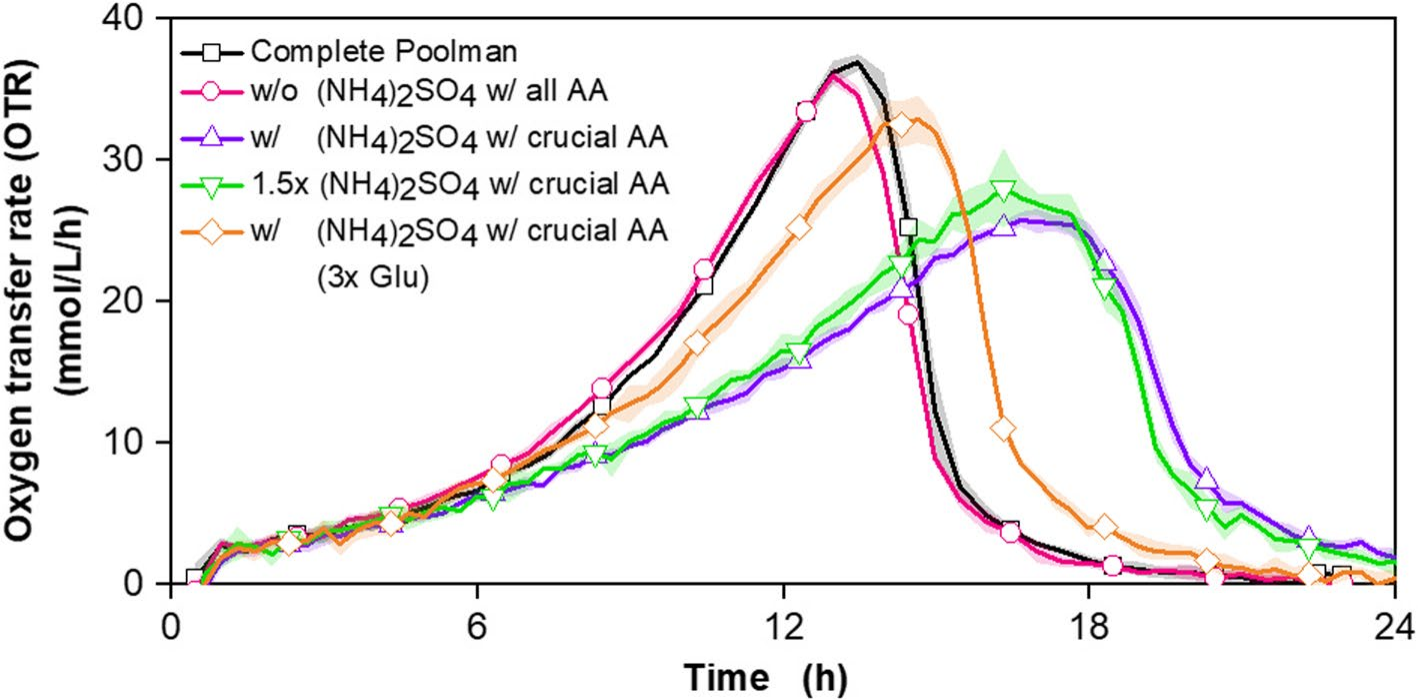
Cultivation of *G. oxydans* 621H Δ*hsdR* pBBR1p264-FDH-ST in a μRAMOS device with 60 g/L fructose with different ammonium and amino acid concentrations. Depicted is the oxygen transfer rate (OTR). Cultivations were performed at 30 °C, 1000 rpm, V_L_ = 500 μL in a 48-well round well microtitre plate at a shaking diameter of 3 mm, initial pH value: 6. AA: amino acids, crucial AA: glutamate, glycine and isoleucine. Media (with elemental nitrogen concentration): Complete Poolman medium as reference (black, 912 mg_N_/L) and without (NH_4_)_2_SO_4_ with all amino acids (pink, 700 mg_N_/L), with (NH_4_)_2_SO_4_ with crucial amino acids (purple, 345 mg_N_/L), with 1.5-fold (NH_4_)_2_SO_4_ with crucial amino acids (green, 443 mg/L N), with (NH_4_)_2_SO_4_ with crucial amino acids with tripled glutamate concentration (orange, 440 mg_N_/L). Mean values of at least 4 replicates are shown. The shadows around the curves indicate the standard deviation. For clarity, only every fifth measuring point is marked as a symbol

After omitting the majority of amino acids from the complete Poolman medium, only 37.8% of the original elemental nitrogen is left. Therefore, it has to be investigated, if the nitrogen requirements of *G. oxydans fdh* are provided in the reduced version of the medium. For that purpose, five cultivations were conducted with different concentrations of (NH_4_)_2_SO_4_ and amino acids to investigate the changes in nitrogen concentration and nitrogen source (Fig. 3). As a reference cultivation, the complete Poolman medium with a concentration of 912 mg_N_/L was used. The second cultivation was conducted without supplementation of (NH_4_)_2_SO_4_ with all amino acids in the original concentration (700 mg_N_/L). In the third cultivation, (NH_4_)_2_SO_4_ and the three crucial amino acids isoleucine, glutamate and glycine were supplemented in the original concentrations (345 mg_N_/L). In cultivation four, a 1.5-fold (NH_4_)_2_SO_4_ concentration was used, while the glutamate concentration was tripled for the fifth cultivation (440 mg_N_/L each).

The cultivation in the complete Poolman medium showed the same OTR course as in previous experiments. The omission of (NH_4_)_2_SO_4_ from the medium did not influence the respiration, while reducing the amino acids to isoleucine, glutamate and glycine shifted the peak to 25 mmol/L/h at 18 hours. A 1.5-fold increase in (NH_4_)_2_SO_4_ concentration did not change the OTR. However, increasing the glutamate concentration shifted the OTR peak back to 35 mmol/L/h at 15 hours.

The omission of (NH_4_)_2_SO_4_ from the medium in cultivation two did not impact the growth of *G. oxydans fdh,* as the same respirational activity was achieved as with the complete Poolman medium. Since (NH_4_)_2_SO_4_ only acts as a nitrogen source, it is not essential for growth, when a sufficient amount of nitrogen is supplied via amino acids. The remaining amount of nitrogen of 700 mg_N_/L exceeds the necessary amount for the growth of *Gluconobacter* strains reported by Sainz et al. [28] (< 300 mg_N_/L) and Hahn et al. [23] (38 mg_N_/L). Moreover, amino acids can act as the sole nitrogen source for various *Gluconobacter* strains [23, 24]. In cultivation three, the growth of *G. oxydans fdh* was negatively impacted, as evidenced by the shift in the OTR. The nitrogen from the three crucial amino acids and (NH_4_)_2_SO_4_ amounts to 345 mg_N_/L, which exceeds the reported necessary concentrations [23, 28]. Since increasing the nitrogen concentration with additional supplementation of (NH_4_)_2_SO_4_ does not improve the growth of *G. oxydans fdh,* a nitrogen limitation is not the cause for the impaired growth. Increasing the glutamate concentration, however, improved the growth of *G. oxydans fdh*, resulting in a higher OTR peak and shorter cultivation time. Because of the importance of glutamate as a precursor for many other amino acids and the cell’s nitrogen supply [46], the demand for glutamate is increased, when only the crucial amino acids are supplemented to the medium. Consequently, the impaired growth is caused by the limited availability of glutamate. Increasing the glutamate concentration in the final medium more than three times, as well as increasing the concentrations of isoleucine and glycine two times, showed no positive influence on the cultivation of *G. oxydans fdh* (Fig. S2). As a result, the glutamate concentration is tripled in the final minimal medium. (NH_4_)_2_SO_4_ remains in the medium at the original concentration of 1 g/L as an additional nitrogen source.

### Determination of essential vitamins for growth of *G. oxydansfdh*

**Fig. 4:**
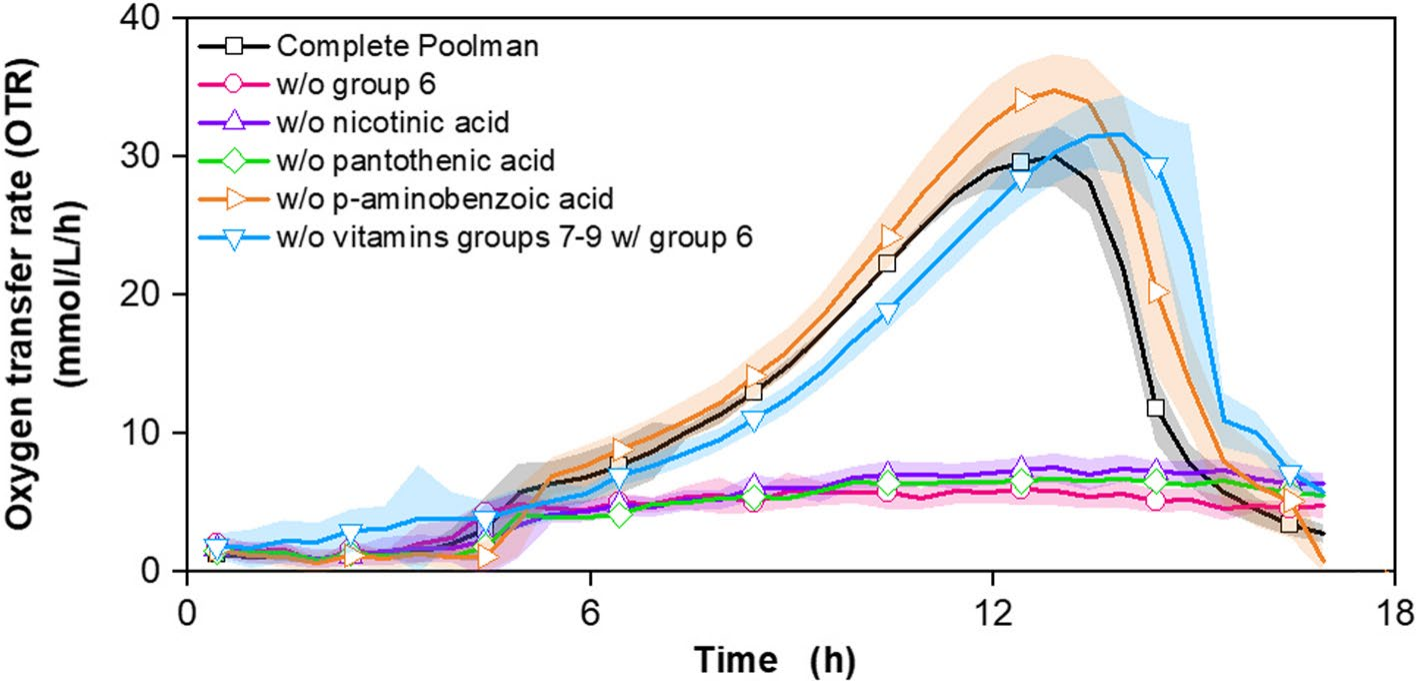
Investigation of auxotrophic deficiencies of *G. oxydans* 621H Δ*hsdR* pBBR1p264-FDH-ST in Poolman medium regarding the vitamins of group 6 (nicotinic acid, pantothenic acid and p-aminobenzoic acid). Depicted is the oxygen transfer rate (OTR). Cultivation of *G. oxydans* 621H Δ*hsdR* pBBR1p264-FDH-ST in a μRAMOS device with 60 g/L fructose, lacking vitamins from group 6 (nicotinic acid, pantothenic acid and p-aminobenzoic acid). Cultivations were performed at 30 °C, 1000 rpm, V_L_ = 500 μL in a 48-well round well microtitre plate at a shaking diameter of 3 mm, initial pH value: 6. Media: Complete Poolman medium as reference (black) and without group 6 (pink), without nicotinic acid (purple), without pantothenic acid (green), without p-aminobenzoic acid (orange) and without vitamins groups 7–9 with group 6 (light blue). Mean values of at least 3 replicates are shown. The shadows around the curves indicate the standard deviation. For cultivation without p-aminobenzoic acid (orange) the mean value of duplicates is shown. For clarity, only every fifth measuring point is marked as a symbol

In this study, 12 vitamins were examined, divided into groups like the amino acids (Table 2). Group 6 consists of the three vitamins nicotinic acid, pantothenic acid and p-aminobenzoic acid, which have already been studied by Underkofler et al. [26] and Gosselé et al. [53]. Group 7 consisted of pyridoxamine and pyridoxine, group 8 of ascorbic acid, biotin, folic acid, orotic acid and riboflavin, and group 9 of thiamine and vitamin B12. First, all groups were removed individually from the complete Poolman medium, investigating their impact on the OTR kinetics. Cultivations without groups 7 and 9 led to a shift of the OTR maximum by about 1 hour, while group 8 showed no influence (Fig. S3). It can be concluded that the components of groups 7 to 9 are not essential Fig. 4 shows the investigation of group 6. When this group is omitted from the complete Poolman medium, a strongly reduced OTR course can be observed. It can be concluded that group 6 contains essential components for the growth of *G. oxydans fdh*. The slight OTR increase of the experiment without group 6 was due to the transfer of media components from pre-culture and the highly active fructose dehydrogenase, as described before [14, 15]. The individual examination of the components of group 6 showed the same decreased OTR course when nicotinic acid and pantothenic acid were removed separately from the complete Poolman medium, indicating both vitamins are essential. Underkofler et al. [26] described pantothenic acid, p-aminobenzoic acid and nicotinic acid as essential vitamins for the growth of *Acetobacter suboxydans*. These vitamins, among others, have been used in the cultivation of *G. oxydans* in recent decades [21, 23, 25, 27, 54, 55]. Gosselé et al. [53] examined 95 *Gluconobacter* strains with regard to pantothenic acid and nicotinic acid. The results showed that 58% of the strains required pantothenic acid and 28% pantothenic acid and nicotinic acid. The growth factors were strain-specific and showed no correlation with subspecies in the genus.

Nicotinic acid is used in metabolism for the synthesis of nicotinamide adenine dinucleotide (NAD^+^). NAD^+^ is essential for glycolysis and the citric acid cycle as well as for the regeneration of ATP [33, 56, 57]. NAD^+^ can be synthesised either de novo from the amino acids aspartate or tryptophan or from the vitamin nicotinic acid [56–58]. As shown in this study, growth of *G. oxydans fdh* is possible without aspartate and tryptophan. It can, therefore, be concluded that *G. oxydans fdh* mainly uses nicotinic acid for NAD^+^ synthesis. Pantothenic acid is a precursor for the synthesis of coenzyme A (CoA), an acyl carrier in fatty acid metabolism. As an important cofactor, CoA plays a major role in the oxidation of pyruvate to acetyl-CoA in the citric acid cycle [33, 59, 60]. CoA can be synthesised de novo from aspartate, alpha-ketovalerate, cysteine and ATP with pantothenic acid as an intermediate [59, 60]. Since the removal of pantothenic acid alone causes a strong decrease in OTR, pantothenic acid cannot be synthesised de novo in our strain and is, therefore, an essential vitamin. The removal of p-aminobenzoic acid from the complete Poolman medium showed no difference in OTR from the reference medium within the standard deviation. Many synthetic media for *G. oxydans* are supplemented with p-aminobenzoic acid [21, 26, 27, 55]. According to Ameyama [47], p-aminobenzoic acid is not an essential vitamin for this organism, as confirmed in this study. It is biosynthetically derived from the pentose phosphate pathway and is, together with glutamate, a precursor for the synthesis of tetrahydrofolate [61, 62]. Tetrahydrofolate is an important cofactor for amino acid and nucleic acid metabolism [62]. Fig. S4 shows a cultivation using a medium containing only the necessary components of the Poolman medium for the cultivation of *G. oxydans fdh* as defined in this study. The addition of p-aminobenzoic acid resulted in an increase in the maximum OTR as well as cell concentration.

In conclusion, it was demonstrated that nicotinic acid and pantothenic acid are essential vitamins for the growth of *G. oxydans fdh*. P-aminobenzoic acid is not an essential vitamin, but increases biomass formation. Thus, all components necessary for the cultivation of *G. oxydans fdh* and production of 5KF have been identified, and the ‘*Gluconobacter* minimal medium’ (GMM) was successfully developed.

### Cultivation performance in *Gluconobacter* minimal medium (GMM) and comparison with different *Gluconobacter* media

**Fig. 5:**
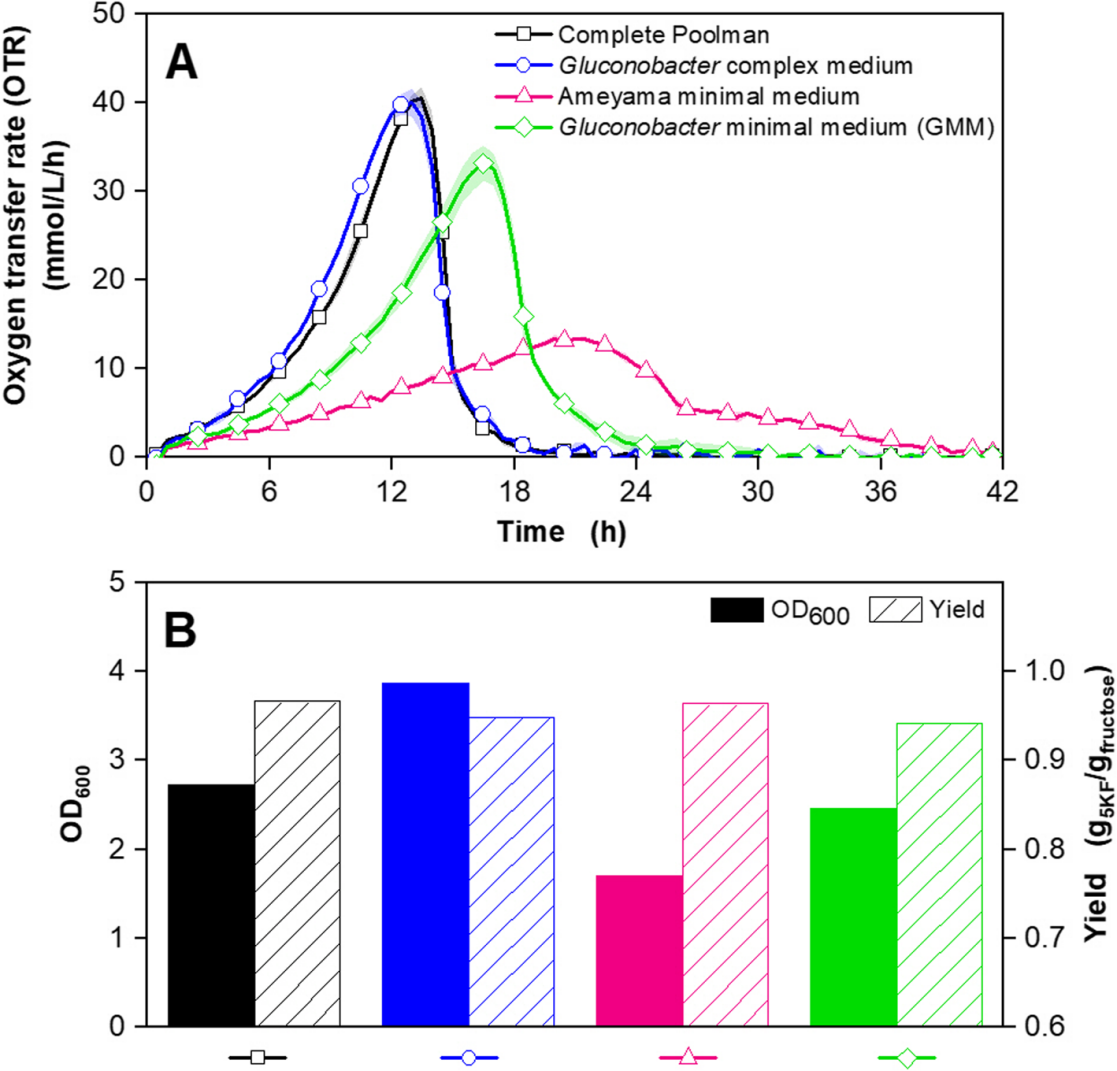
Cultivation of *G. oxydans* 621H Δ*hsdR* pBBR1p264-FDH-ST in a μRAMOS device with 70 g/L fructose and different media. Depicted is **A** the oxygen transfer rate (OTR) and **B** the optical density OD_600_ (solid bars) and the product yield g_5KF_/g_fructose_ (hatched bars). Cultivations were performed at 30 °C, 1000 rpm, V_L_ = 500 μL in a 48-well round well microtitre plate at a shaking diameter of 3 mm, initial pH value: 6. Media: Complete Poolman medium as reference (black), *Gluconobacter* complex medium containing 5 g/L yeast extract, 2.5 g/L MgSO_4_ ∙ 7H_2_O, 1 g/L (NH_4_)_2_SO_4_ and 1 g/L KH_2_PO_4_ (blue), Ameyama minimal medium (pink) and *Gluconobacter* minimal medium (GMM, green), developed in this work. In **A** mean values of at least 3 replicates and in **B** mean values of duplicates are shown. The shadows around the curves indicate the standard deviation. For clarity, only every fifth measuring point is marked as a symbol in **A**

To demonstrate the successful development of a minimal medium for the growth of *G. oxydans fdh*, the GMM developed in this work, the complete Poolman medium, the *Gluconobacter* complex medium and the medium described by Ameyama et al. [27] were compared (Table 1). Figure 5 shows a comparison of cultivations with the four different media. Fructose was completely consumed in all cultivations. Initial fructose concentrations and final 5KF concentrations are displayed in Fig. S5. The cultivation in the complete Poolman medium reached a maximum OTR of approx. 40 mmol/L/h after 13 hours. The cultivation in the complex medium showed a similar OTR course (Fig. 5 A), while showing slight differences in optical density and product yield (Fig. 5 B). Compared to Fig. 1, the OTR maximum reached during the cultivation in complex medium and complete Poolman medium was reached 6 hours and 1.5 hours earlier, respectively. As described before in numerous studies, the chemical composition of yeast extract, commonly used in complex media, is undefined and can dramatically vary depending on the production process [2, 3, 63]. This is confirmed in the presented results. A chemically defined medium consists of pure chemicals with known concentrations. The cultivation in complex medium achieved an OD_600_ of 3.9, which is higher than in complete Poolman medium with an OD_600_ of approx. 2.8 (Fig. 5 B). It is well known that *G. oxydans* generally achieves only low growth rates and biomass yields [14, 33, 64]. However, yields of more than 0.95 g_5KF_/g_fructose_ were achieved in both cultivations. Looking at the stoichiometry of the 5KF production, it becomes apparent that only a small part of the fructose is used for biomass production [14]. As result, the process in the complete Poolman medium is well comparable to cultivation in the complex medium, despite small differences in biomass formation. Thus, the complete Poolman medium with its 48 components (Table 1) was an optimal starting point for the development of a minimal medium for the cultivation of *G. oxydans fdh* (Fig. 5 A).

In this work, the complete Poolman medium was reduced by more than two thirds to 17 components and named GMM. The comparison of the OTR curves shows that the cultivation in the GMM started with lower growth rate, and a lower maximum OTR was reached (Fig. 5 A). Overall, the cultivation time of 30 hours is 6 hours longer than the cultivation in the complete Poolman medium. This is not surprising, as *G. oxydans fdh* has to synthesize the missing components itself. The OD_600_ is only 0.2 lower than in the complete Poolman medium. This is remarkable since only 17 of the 48 components of the Poolman medium are included in the GMM. Moreover, a yield of 0.94 g_5KF_/g_fructose_ was achieved. This yield is in the same range as for complex medium and complete Poolman medium. It shows that the systematic identification of the necessary components for the growth of *G. oxydans fdh* and the production of 5KF was successful. In order to exclude the possibility that media components from the pre-culture cultivation in complex medium were transferred to the main cultivation with GMM, a second pre-culture in GMM was carried out in one experiment. The results displayed in Fig. S6 show that the second pre-cultivation medium in GMM has no negative influence on the main culture in GMM.

Finally, a comparison is to be made with a minimal medium for *Gluconobacter* described in the literature. Therefore, the modified medium according to Ameyama et al. [27] has been included for comparison as a representative of defined media. Despite great similarities in the media composition, as displayed in Table 1, the experiment showed that the OTR curve of the OTR course of the cultivation in the Ameyama medium is very flat and only reached a maximum of 15 mmol/L/h after 20 hours. The total cultivation time is prolonged to 42 hours. There are two main differences between the Ameyama medium and the GMM. First, the Ameyama medium only contains two trace elements, FeSO_4_ and MnSO_4_. Trace elements are essential components of microbial growth and must be supplemented [38, 65, 66]. As these components are only needed in very low concentrations, they could also have been transferred from the pre-culture to the main culture and should not influence growth. Second, the Ameyama medium contains only one amino acid, glutamate. It has already been demonstrated in this study that a systematical supplementation of amino acids has a considerable influence on the growth of *G. oxydans*. As depicted in Fig. 5 B, the OD_600_ of the cultivation in the Ameyama medium is 0.8 lower than in the GMM medium. In conclusion, a minimal medium for the cultivation of *G. oxydans fdh* and 5KF production containing 17 ingredients, including three amino acids, three vitamins, trace elements and the main ingredients MgSO_4_ ∙ 7H_2_O, (NH_4_)_2_SO_4_, KH_2_PO_4_ (Table 1), was successfully developed.

### 5KF fermentation in 2 L fermenter using GMM

**Fig. 6:**
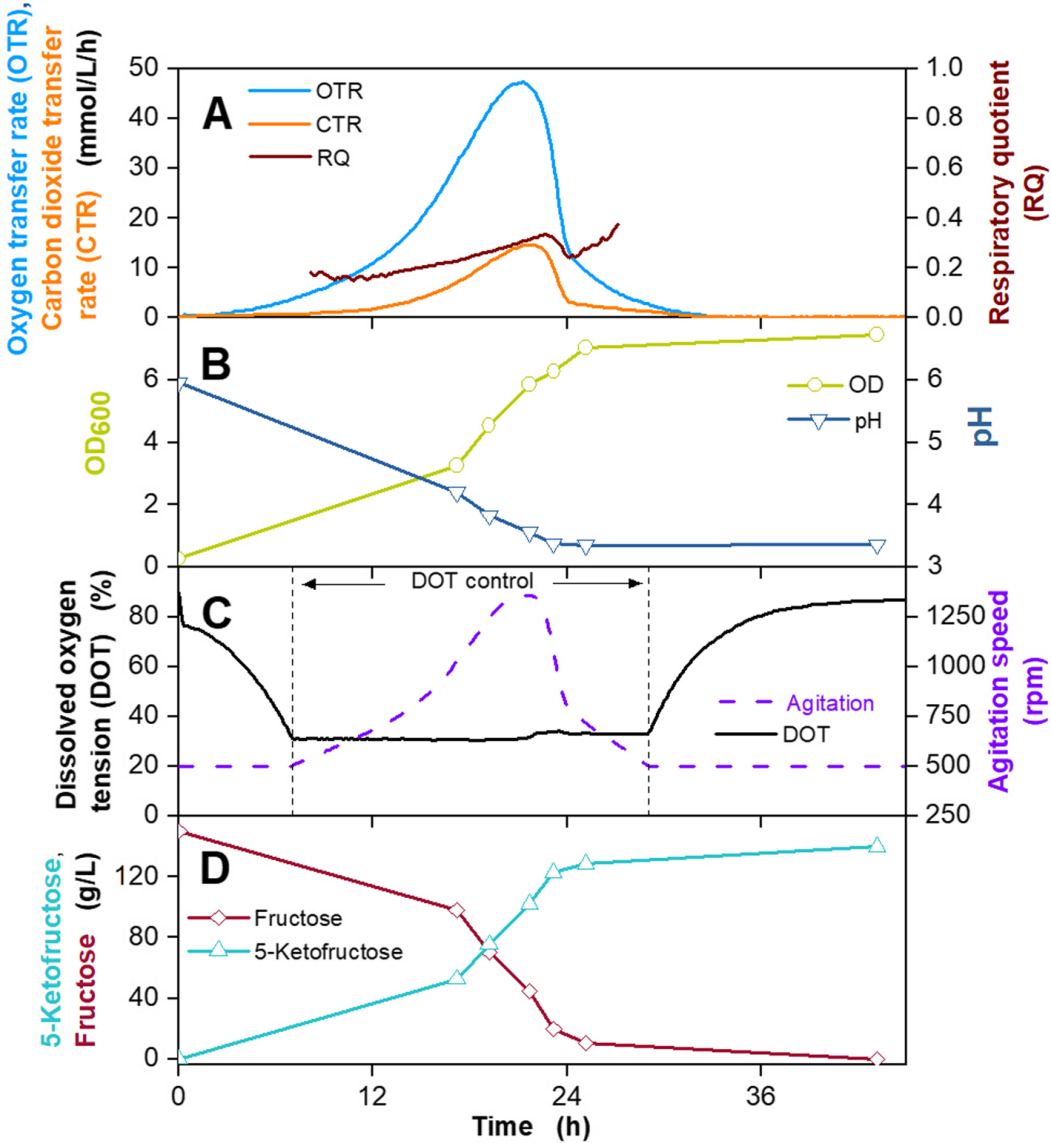
Batch cultivation of *G. oxydans* 621H Δ*hsdR* pBBR1p264-FDH-ST in a 2 L fermenter in *Gluconobacter* minimal medium (GMM), developed in this work. Depicted is **A** the oxygen transfer rate (OTR, light blue), carbon dioxide transfer rate (CTR, orange) and respiratory quotient (RQ, brown), **B** the optical density OD_600_ (olive) and pH (dark blue), **C** the dissolved oxygen tension (DOT, black) and agitation speed (purple), **D** fructose (dark red) and 5-ketofructose concentration (turquoise). Cultivation was performed in *Gluconobacter* minimal medium (GMM), with 150 g/L fructose at 30 °C, initial pH value 6, no pH control, V_L_ = 1 L in a 2 L fermenter. DOT was kept ≥30% by variation of the agitation speed (500–1350 rpm), absolute aeration rate Q_g_ = 1 L/min. RQ-values are only shown, when OTR-values are above 5 mmol/L/h

The development of the GMM was carried out using the production of 5KF as an exemplary aim. 5KF production was described by Herweg et al. [14] and is based on the *Gluconobacter* complex medium [15, 67]. The initial fructose concentration of the 5KF production process is 150 g/L. Hence, a first batch fermentation in a stirred 2 L fermenter was performed using GMM and 150 g/L fructose (Fig. 6). The OTR reaches a maximum of 48 mmol/L/h after approx. 20 hours. The CTR shown in Fig. 6 A is lower than the OTR, because 5KF production is stoichiometrically coupled to oxygen consumption. The RQ is in the range between 0.2 and 0.3. This is typical for 5KF production and indicates high product formation [14, 67]. Due to the uptake of ammonium, the pH drops from 6.0 to 3.5 during cultivation, as no buffer or pH control was applied [14, 67]. The FDH has a pH optimum at 4 and the activity decreases strongly at lower pH values, leading to a decreasing OTR after approx. 20 h [68]. The OD_600_ rises to approx. 7 after 24 hours. In order to regulate the oxygen consumption at 30%, the agitation speed was increased to 1300 rpm in the period between 6 and 30 hours. The 150 g/L fructose was entirely consumed in about 30 hours, and 140 g/L 5KF was produced. The resulting product yield was 0.93 g_5KF_/g_fructose_. Compared to the batch fermentation of Herweg et al. with *G. oxydans fdh* in *Gluconobacter* complex medium [14], comparable results for on- and offline values were obtained.

**Fig. 7:**
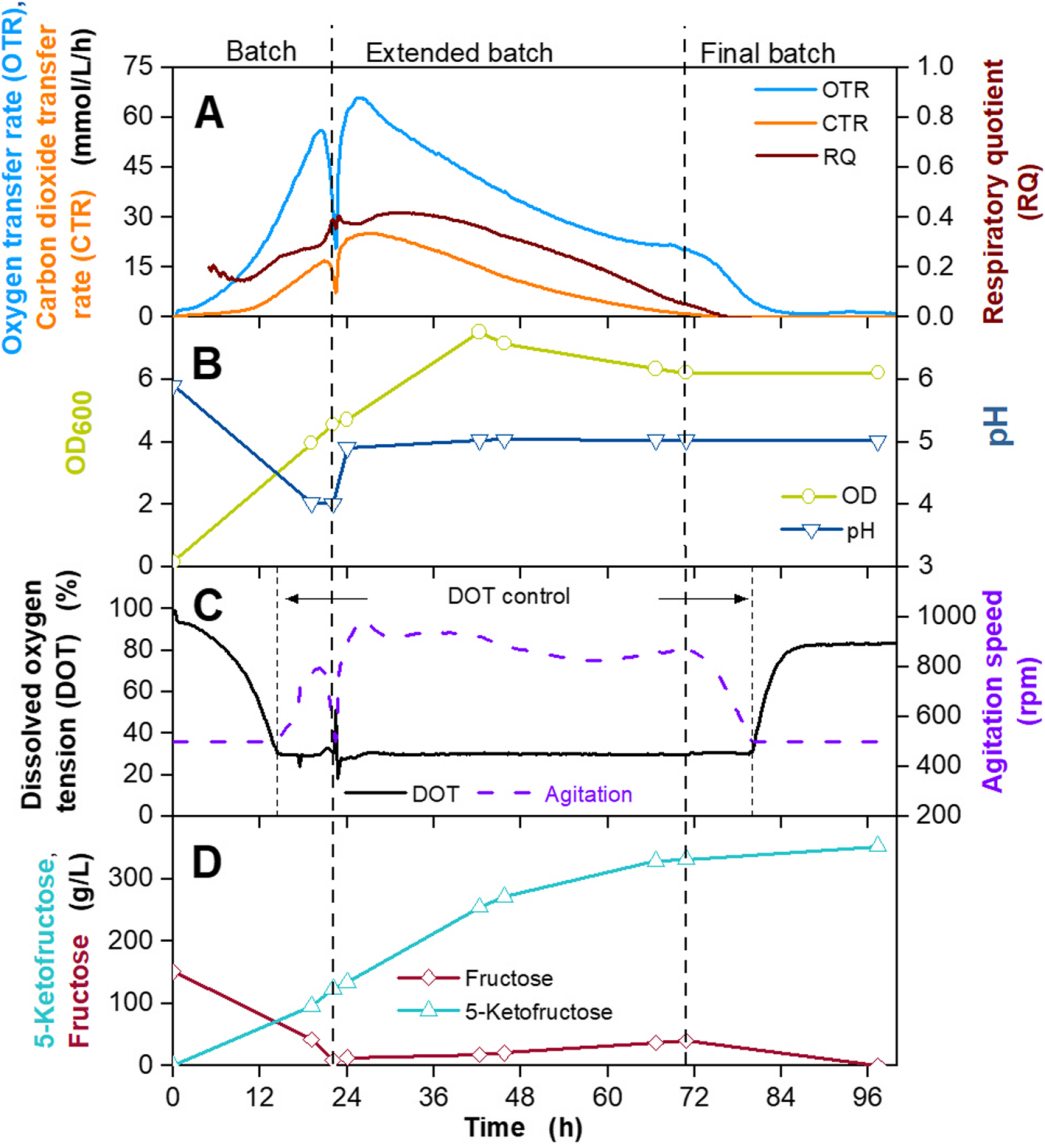
Extended batch cultivation of *G. oxydans* 621H Δ*hsdR* pBBR1p264-FDH-ST in a 2 L fermenter in *Gluconobacter* minimal medium (GMM) developed in this work. Depicted is A the oxygen transfer rate (OTR, light blue), carbon dioxide transfer rate (CTR, orange) and respiratory quotient (RQ, brown), B the optical density OD_600_ (olive) and pH (dark blue), C the dissolved oxygen tension (DOT, black) and agitation speed (purple), D fructose (dark red) and 5-ketofructose concentration (turquoise). Cultivation was performed in *Gluconobacter* minimal medium (GMM) with 150 g/L fructose at 30 °C, initial pH value 6, pH control at 5 from 22 h with 10 M KOH, V_L,start_ = 1 L in a 2 L fermenter. DOT was kept ≥30% by variation of the agitation speed (500–1000 rpm), absolute aeration rate Q_g_ = 1 L/min. Feeding solution: 825 g_fructose_/L, Feed rate: 15.5 g_fructose_/h, t_feed_ = 22–71 h. RQ-values are only shown, when OTR-values are above 5 mmol/L/h

In a final step, the GMM medium was tested in an extended batch fermentation. Herweg et al. [14] described the development of the 5KF production process. The fermentation is divided into three parts: a batch phase, followed by an extended batch with constant fructose feeding, and a final batch. In this study, two extended batch fermentations were performed using GMM and different fructose feeding rates (Fig. 7 and Fig. S7). Regarding the batch phase of both fermentations, similar results were achieved, compared to the batch fermentation shown in

Figure 6 the OTR reached a local maximum of approx. 50–55 mmol/L/h, the OD_600_ increased to 4.5–5.0, and pH decreased to 4. This confirms the excellent reproducibility of the GMM medium during 5KF production. After 20 hours, the OTR started decreasing, due to the exhaustion of fructose (Fig. 7 B). Hence, fructose feeding and pH control at a pH of 5 was started. The FDH has a relative activity of 100% at a pH of 4, which slightly decreases to 70% at a pH of 5 [68]. The growth optimum of *G. oxydans* 621H is at a pH between 5.5 and 6 [55]. To promote both, 5KF production and cell gowth, pH was maintained at 5. During the extended batch phase displayed in Fig. 7, a fructose feed rate of 15.5 g_fructose_/h was applied, using an 825 g_fructose_/L solution. In order to regulate the oxygen consumption at 30%, the agitation speed was increased to a maximum value of 1000 rpm. OTR and CTR increased for the first 6 hours of this phase, verifying high cell growth and 5KF production, as also OD_600_ and 5KF concentration increased. After approx. 24 h, OTR and CTR start to decrease despite sufficient substrate and oxygen supply. This is supposedly connected to a negative influence of increasing product concentrations and elevated osmolality. This phenomenon will be described in detail in another work (manuscript in preparation). At 72 h, the CTR reached 0 mmol/L/h, indicating the end of cell growth, as no carbon dioxide was formed anymore. Feeding was stopped at this time. During the extended batch phase, fructose concentration remained between 10 and 40 g_fructose_/L. The residual fructose concentration was converted to 5KF during the final batch, and a 5KF titre of 350 g_5KF_/L was reached, with a yield of 0.75 g_5KF_/g_fructose_ and an overall productivity of 3.8 g_5KF_/L/h.

The extended batch fermentation displayed in Fig. S7 showed similar shaped OTR and CTR curves, compared to Fig. 7. The DOT showed fluctuations between 12 and 24 h, which were caused by technical errors of the pO2-sensor and did not influence the cultivation. During the extended batch phase displayed in Fig. S7, a higher fructose feed rate of 23 g_fructose_/h was applied using a 770 g_fructose_/L solution. The increased feed rate, compared to Fig. 7, led to the accumulation of fructose during the extended batch phase. As shown in a previous publication, high fructose concentrations above 150 g/L can negatively influence growth of *G. oxydans* fdh [14]. A decreased fructose feed rate as used in Fig. 7 prevented fructose accumulation. Despite fructose accumulation, a final 5KF titre of 355 g_5KF_/L, a yield of 0.81 g_5KF_/g_fructose_ and a productivity of 4.2 g_5KF_/L/h was reached (Fig. S7). Overall, similar results were obtained, compared to the first extended batch fermentation displayed in Fig. 7, showing the good reproducibility of this process.

## Conclusions

In this work, we successfully developed a minimal medium for cultivation and product formation with *Gluconobacter oxydans* 621H Δ*hsdR* pBBR1-p264-FDH-ST. The resulting *Gluconobacter* minimal medium (GMM) is based on the rich complete Poolman medium that was systematically reduced to the components most important for growth and product formation in *G. oxydans fdh.* Assessment of the influence of various media components on the cultivation was based on respiration data recorded in 48 parallel wells of a microtitre plate with a μRAMOS-device. As a result, the vitamins nicotinic acid and pantothenic acid have been identified as essential for *Gluconobacter fdh*. The vitamin p-aminobenzoic acid and the three amino acids glutamate, isoleucine and glycine have been found to be crucial for an efficient cultivation of *G. oxydans fdh*. Together with trace elements and the main components (carbon, nitrogen, magnesium and phosphate source), the GMM was composed. The GMM performed well compared to the complete Poolman medium and *Gluconobacter* complex medium and showed clearly better growth and production characteristics than a minimal medium from the literature. In a final step, the GMM was tested in laboratory-scale fermentation in extended batch mode, where 5KF titres of up to 355 g/L and productivities of 4.2 g_5KF_/L/h were observed. Compared with the fermentation data published by Herweg et al. [14], the 5KF titre, productivity and yield were lower using GMM. Lower concentrated fructose feeding solutions resulted in a dilution of the fermentation broth. Possibilities for optimisation include adjustment of single media components concentrations and further development of the extended batch fermentation process for 5KF production using the chemically defined GMM. Nevertheless, the detailed knowledge of components in the fermentation broth can improve downstream processing, as complex media components can complicate downstream processing [1, 4–6]. In summary, the GMM is suited as a replacement for the *Gluconobacter* complex medium for 5KF production.

On top of the systematic development of a new minimal medium for *G. oxydans fdh,* an in-depth understanding of the influence of various media components on the metabolism of *G. oxydans fdh* was gained, which can be transferred on other production processes with *Gluconobacter strains*. In the future, the developed GMM can help to improve reproducibility and reduce costs of bioproduction processes with *G. oxydans*, by facilitating easier downstream processing and thereby contribute to a sustainable bioeconomy.

## Supplementary Information

**Additional file 1.**

## Data Availability

The datasets supporting the conclusions of this article are included within the article and the additional file (Additional file 1.pdf: Figs. S1, S2, S3, S4, S5, S6 and S7).

## Abbreviations

5KF: 5-keto-d-fructose
ADP: adenosine diphosphate
ATP: Adenosine triphosphate
CoA: Coenzyme A
CTR: Carbon dioxide transfer rate
DOT: Dissolved oxygen tension
FDH: Fructose dehydrogenase
GMM: Gluconobacter minimal medium
HPLC: High-performance liquid chromatography
MTP: Microtitre plate
NAD^+^: Nicotinamide adenine dinucleotide
OD_600_: Optical density at 600 nm
OTR: Oxygen transfer rate
RAMOS: Respiration activity MOnitoring System
RQ: Respiratory quotient
