# Supplementary material for: Development of a novel defined minimal medium for Gluconobacter oxydans 621H by systematic investigation of metabolic demands
Source: J Biol Eng. 2022 Nov 21;16:31. doi: 10.1186/s13036-022-00310-y (PMC9682679; doi:10.1186/s13036-022-00310-y)
Supplement: Supplementary file 1 — Additional file 1. [file 13036_2022_310_MOESM1_ESM.docx]

# Additional file 1

**Development of a novel defined minimal medium for Gluconobacter oxydans 621H by systematic investigation of metabolic demands**

Svenja Battling**, Johannes Pastoors**, Alexander Deitert, Tobias Götzen, Lukas Hartmann, Eliot Schröder, Stanislav Yordanov and Jochen Büchs*

AVT-Chair for Biochemical Engineering, RWTH Aachen University, Forckenbeckstraße 51, 52074 Aachen, Germany

*Correspondence: [jochen.buechs@avt.rwth-aachen.de](mailto:jochen.buechs@avt.rwth-aachen.de)

**Both authors contributed equally to this manuscript

**Fig. S1**. Investigation of auxotrophic deficiencies of *G. oxydans* 621H Δ*hsdR* pBBR1p264-FDH-Strep in Poolman medium regarding the amino acids.

**Fig. S2**. Cultivation of *G. oxydans* 621H Δ*hsdR* pBBR1p264-FDH-Strep in a µRAMOS device with 60 g/L fructose in *Gluconobacter* minimal medium with different ammonium sulfate and amino acid concentrations.

**Fig. S3**. Cultivation of *G. oxydans* 621H Δ*hsdR* pBBR1p264-FDH-Strep in a µRAMOS device with 60 g/L fructose, lacking different vitamins.

**Fig. S4**. Cultivation of *G. oxydans* 621H Δ*hsdR* pBBR1p264-FDH-Strep in a µRAMOS device with 60 g/L fructose in *Gluconobacter* minimal medium with and without the addition of p-aminobenzoic acid.

**Fig. S5**. Cultivation of *G. oxydans* 621H Δ*hsdR* pBBR1p264-FDH-Strep in a µRAMOS device with 70 g/L fructose in different media.

**Fig. S6**. Cultivation of *G. oxydans* 621H Δ*hsdR* pBBR1p264-FDH-Strep in a µRAMOS and RAMOS device with 60 g/L fructose in *Gluconobacter* minimal medium (GMM).

**Fig. S7**. Extended batch-cultivation of *G. oxydans* 621H Δ*hsdR* pBBR1p264-FDH-Strep in a 2 L fermenter in *Gluconobacter* minimal medium (GMM).


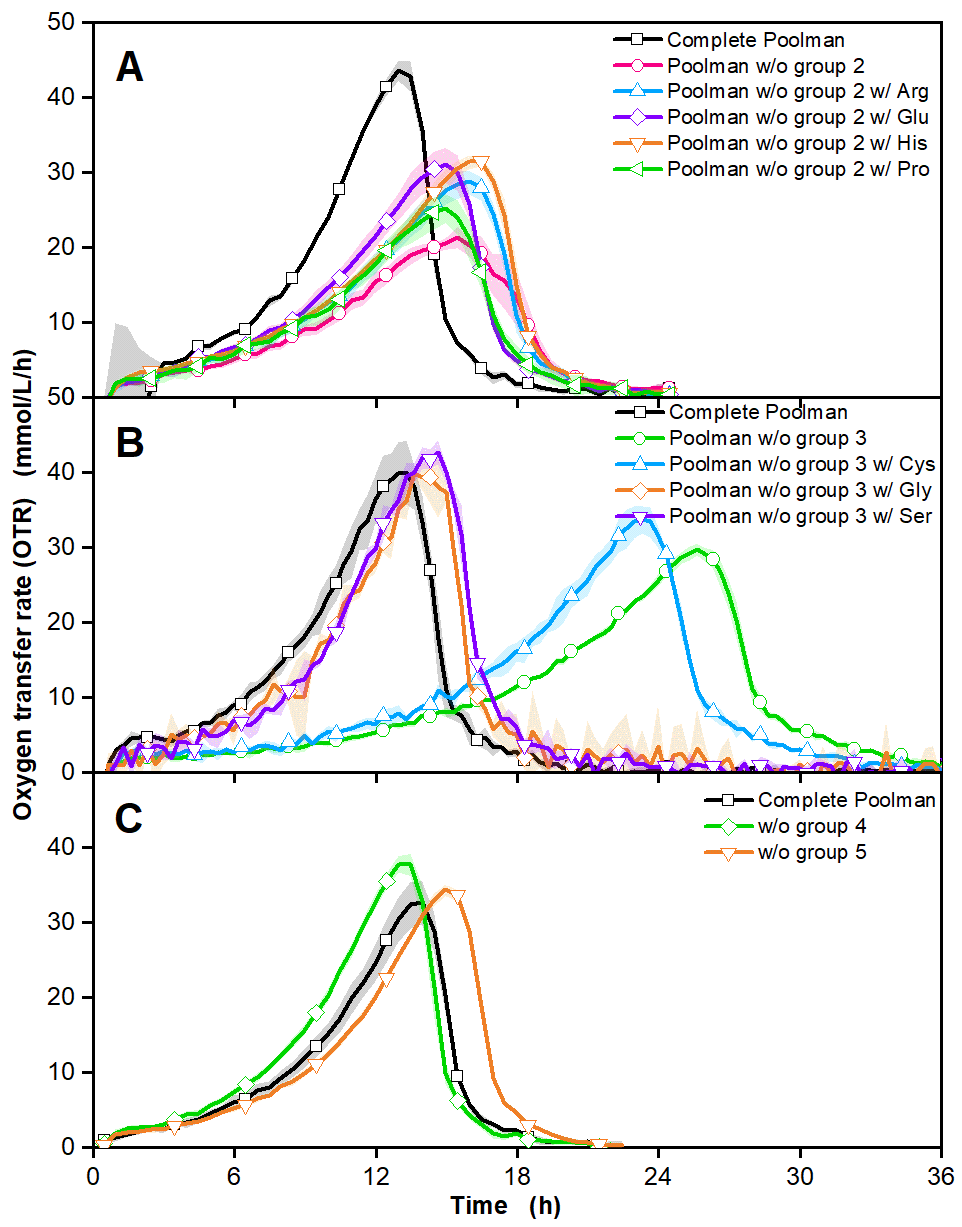


**Fig. S1:** **Investigation of auxotrophic deficiencies of G. oxydans 621H ΔhsdR pBBR1p264-FDH-Strep in Poolman medium regarding the amino acids.** Depicted is the oxygen transfer rate (OTR). Cultivation of *G. oxydans* 621H Δ*hsdR* pBBR1p264-FDH-Strep in a µRAMOS device with 60 g/L fructose, lacking amino acids from group 2 (histidine, arginine, glutamate or proline). Three independent cultivations (**A, B** and **C**) were each performed at 30 °C, 1000 rpm, V_L_ = 500 µL in a 48-well round well microtitre plate at a shaking diameter of 3 mm, initial pH value: 6. Media: Complete Poolman medium as reference (black) and **A** without group 2 (pink), without group 2 with arginine (light blue), without group 2 with glutamate (purple), without group 2 with histidine (orange) and without group 2 with proline (green), **B** without group 3 (green), without group 3 with cysteine (light blue), without group 3 with glycine (orange) and without group 3 with serine (purple) and **C** without group 4 (green) and without group 5 (orange). Mean values of at least 3 replicates are shown. The shadows around the curves indicate the standard deviation. For clarity, only every fifth measuring point is marked as a symbol.


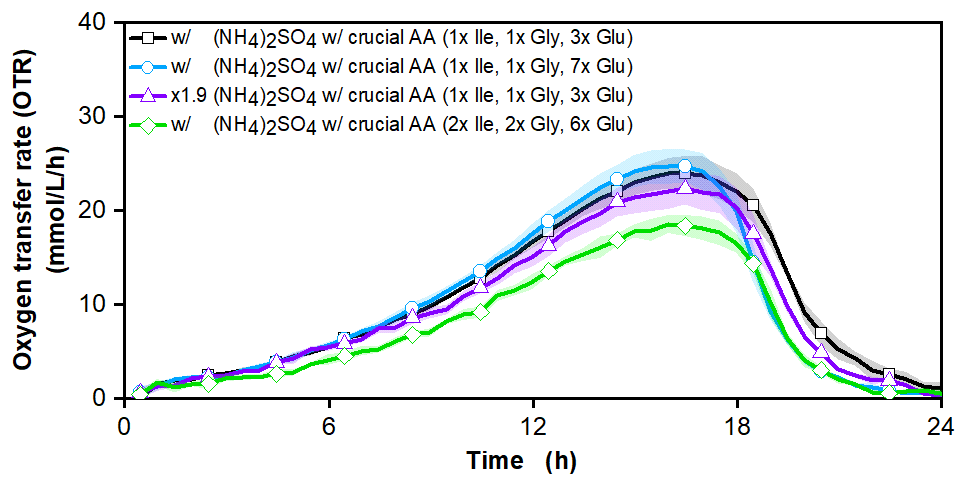


**Fig. S2: Cultivation of *G. oxydans* 621H Δ*hsdR* pBBR1p264-FDH-Strep in a µRAMOS device with 60 g/L fructose in *Gluconobacter* minimal medium with different ammonium sulfate and amino acid concentrations.** Depicted is the oxygen transfer rate (OTR). Cultivations were performed at 30 °C, 1000 rpm, V_L_ = 500 µL in a 48-well round well microtitre plate at a shaking diameter of 3 mm, initial pH value: 6. AA: amino acids, crucial AA: glutamate, glycine and isoleucine. Nitrogen concentrations were adjusted from *Gluconobacter* minimal medium (black, 410 mg_N_/L) to the value of the *Gluconobacter* complex medium (609 mg_N_/L) in different ways: With 7x glutamate (light blue), 1.9 x ammonium sulfate and 3x glutamate (purple), 2x isoleucin, 2x glycin and 6x glutamate (green). Mean values of at least 4 replicates are shown. The shadows around the curves indicate the standard deviation. For clarity, only every fifth measuring point is marked as a symbol.


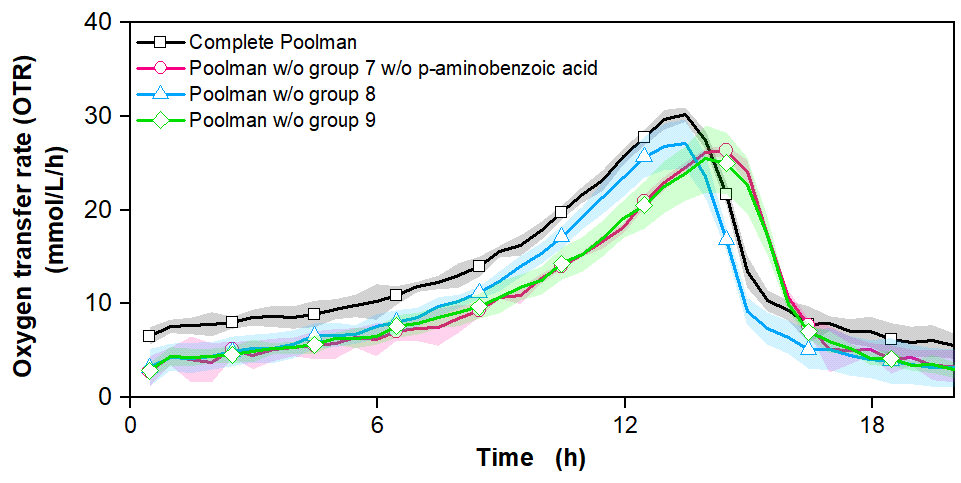


**Fig. S3: Cultivation of *G. oxydans* 621H Δ*hsdR* pBBR1p264-FDH-Strep in a µRAMOS device with 60 g/L fructose, lacking different vitamins.** Depicted is the oxygen transfer rate (OTR). Cultivations were performed at 30 °C, 1000 rpm, V_L_ = 500 µL in a 48-well round well microtitre plate at a shaking diameter of 3 mm, initial pH value: 6. Media: Complete Poolman medium as reference (black) and without group 7 (pyridoxamine, pyridoxine) and with p-aminobenzoic acid (pink), without group 8 (folic acid, ascorbic acid, ortoric acid, riboflavin, biotin) (light blue) and without group 9 (thiamine, vitamin B12) (green). Mean values of at least 3 replicates are shown. The shadows around the curves indicate the standard deviation. For clarity, only every fifth measuring point is marked as a symbol.


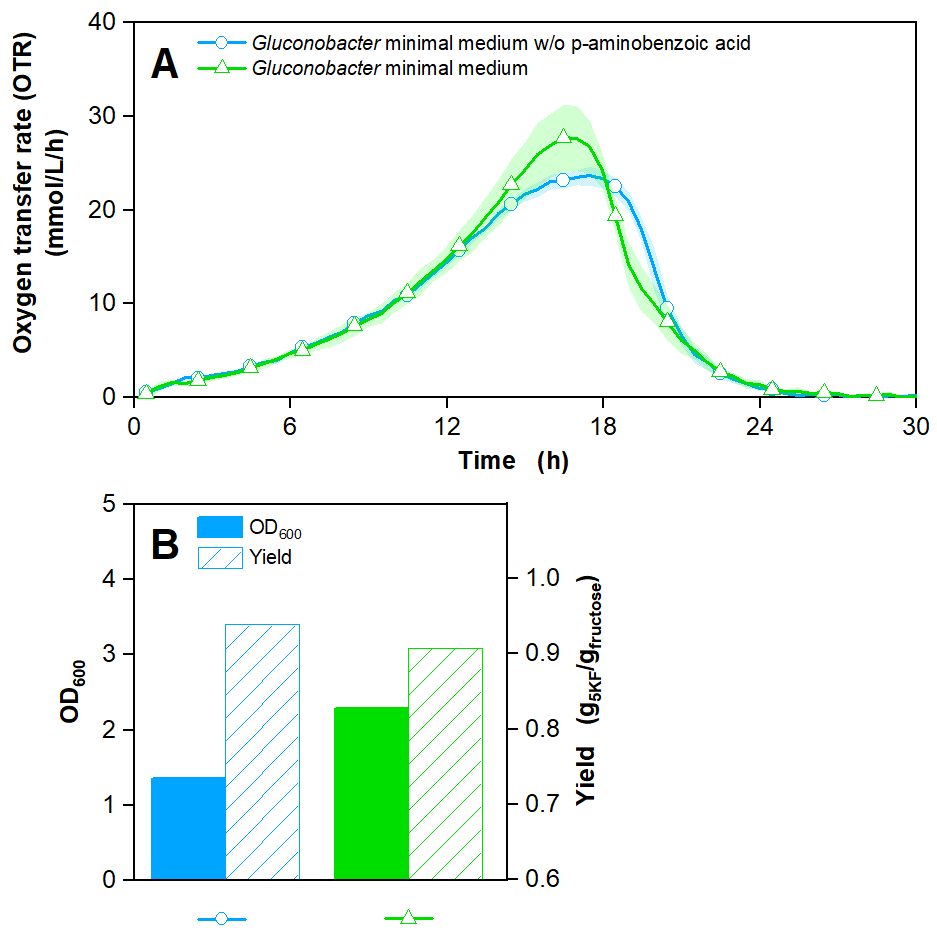


**Fig. S4: Cultivation of *G. oxydans* 621H Δ*hsdR* pBBR1p264-FDH-Strep in a µRAMOS device with 60 g/L fructose in *Gluconobacter* minimal medium with and without the addition of p-aminobenzoic acid.** Depicted is **A** the oxygen transfer rate (OTR) and **B** the optical density OD_600_ (solid bars) and the yield (hatched bars). Cultivations were performed at 30 °C, 1000 rpm, V_L_ = 500 µL in a 48-well round well microtitre plate at a shaking diameter of 3 mm, initial pH value: 6. Media: *Gluconobacter* minimal medium without p-aminobenzoic acid (light blue) and *Gluconobacter* minimal medium (green). Mean values of at least 4 replicates (**A**) and mean values of duplicates (**B**) are shown. The shadows around the curves indicate the standard deviation (**A**). For clarity, only every fifth measuring point is marked as a symbol in **A**.


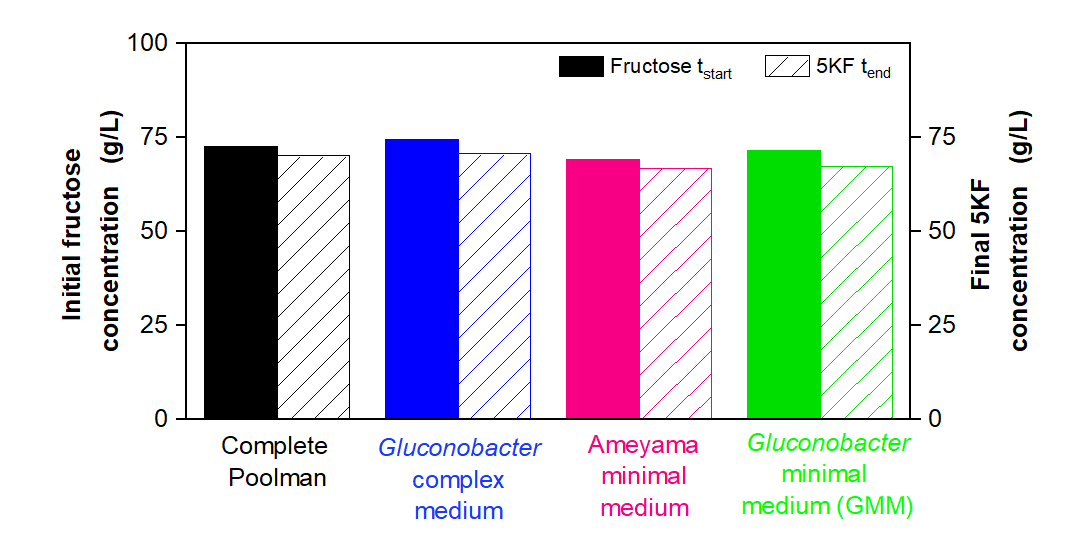


**Fig. S5: Cultivation of *G. oxydans* 621H Δ*hsdR* pBBR1p264-FDH-Strep in a µRAMOS device with 70 g/L fructose in different media.** Depicted is the initial fructose concentration (solid bars) and the final 5KF concentration (hatched bars). Cultivations were performed at 30 °C, 1000 rpm, V_L_ = 500 µL in a 48-well round well microtitre plate at a shaking diameter of 3 mm, initial pH value: 6. Media: Complete Poolman medium as reference (black), *Gluconobacter* complex medium containing 5 g/L yeast extract, 2.5 g/L MgSO_4_∙7H_2_O, 1 g/L (NH_4_)_2_SO_4_ and 1 g/L KH_2_PO_4_ (blue), Ameyama minimal medium (pink) and *Gluconobacter* minimal medium (GMM, green), developed in this work.


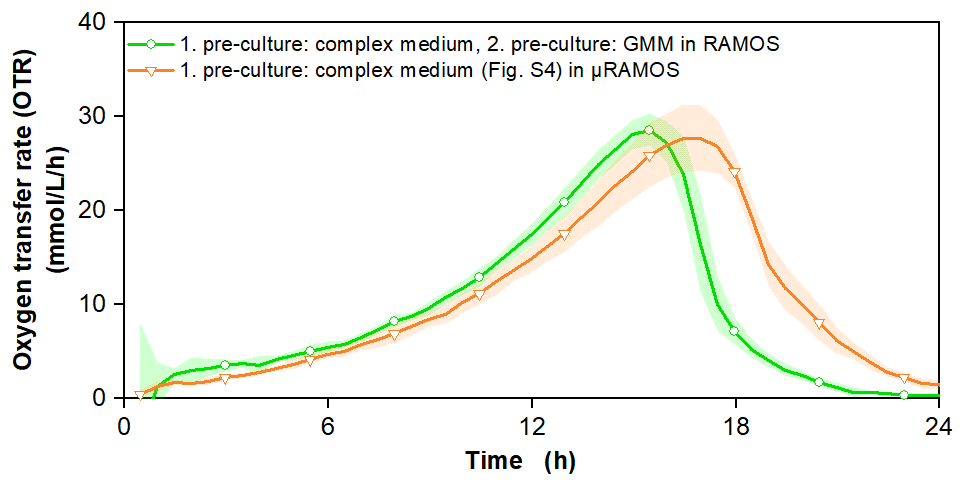


**Fig. S6: Cultivation of *G. oxydans* 621H Δ*hsdR* pBBR1p264-FDH-Strep in a µRAMOS and RAMOS device with 60 g/L fructose in *Gluconobacter* minimal medium (GMM).** Depicted is the oxygen transfer rate (OTR). Cultivations were performed at 30 °C in a µRAMOS device at 1000 rpm, V_L_ = 500 µL in a 48-well round well microtitre plate at a shaking diameter of 3 mm or in a RAMOS device at 350 rpm, V_L_ = 10 mL in 250 mL unbaffled shake flasks at a shaking diameter of 50 mm. Shown are the cultivations in *Gluconobacter* minimal medium (GMM) inoculated from a pre-culture in *Gluconobacter* complex medium from Fig. S4 (orange) in a µRAMOS device and a cultivation in GMM inoculated from a two-step pre-culture, first in *Gluconobacter* complex medium and second in GMM (green) performed in a RAMOS device. Mean values of 4 replicates are shown for µRAMOS cultivation. The shadow around the curve indicates the standard deviation. Mean values of 2 replicates are shown for RAMOS cultivation. The shadow around the curve indicates highest and lowest value of duplicates. For clarity, only every fifth measuring point is marked as a symbol.

**
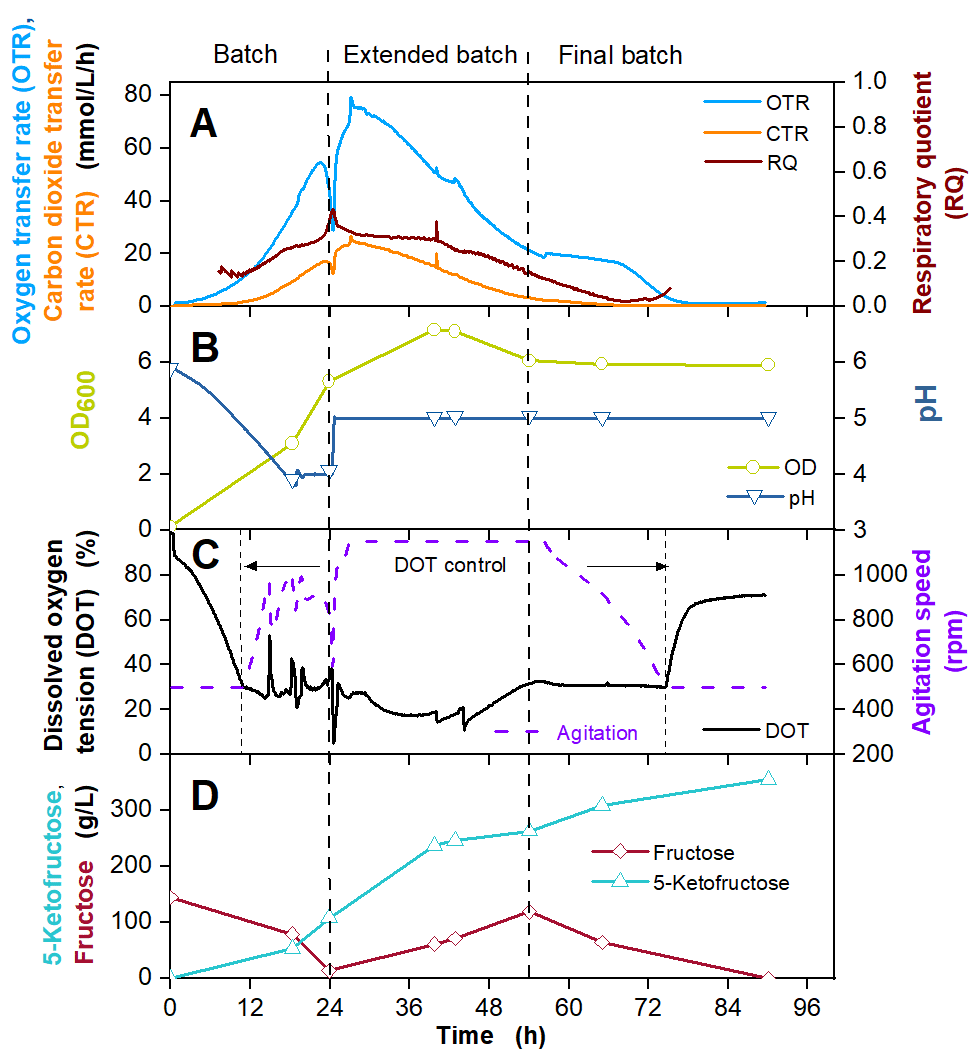
**

**Fig. S7: Extended batch-cultivation of *G. oxydans* 621H Δ*hsdR* pBBR1p264-FDH-Strep in a 2 L fermenter in *Gluconobacter* minimal medium (GMM).** Depicted is **A** the oxygen transfer rate (OTR, light blue), carbon dioxide transfer rate (CTR, orange) and respiratory quotient (RQ, brown), **B** the optical density OD_600_ (olive) and pH (blue), **C** the dissolved oxygen tension (DOT, black) and agitation speed (purple), **D** fructose (dark red) and 5-ketofructose concentration (turquoise). Cultivation was performed in *Gluconobacter* minimal medium with 150 g/L fructose at 30°C, initial pH value: 6, pH control at 5 from 24 h with 10 M KOH, V_L,start_ = 1 L in a 2 L fermenter. DOT was kept ≥ 30 % by variation of the agitation speed (500 – 1350 rpm), absolute aeration rate Q_g_ = 1 L/min. Feeding solution: 770 g_fructose_/L, Feed rate: 23.4 g_Fructose_/h, t_Feed_ = 24 h – 54 h. RQ-values are only shown when OTR-values are above 5 mmol/L/h.
